# AKT/mTOR as Novel Targets of Polyphenol Piceatannol Possibly Contributing to Inhibition of Proliferation of Cultured Prostate Cancer Cells

**DOI:** 10.5402/2012/272697

**Published:** 2012-04-03

**Authors:** Tze-Chen Hsieh, Chia-Yi Lin, Hung-Yun Lin, Joseph M. Wu

**Affiliations:** ^1^Department of Biochemistry & Molecular Biology, New York Medical College, Valhalla, New York 10595, USA; ^2^Genome and Systems Biology Degree Program, National Taiwan University, Taipei 10617, Taiwan; ^3^Signal Transduction Laboratory, Ordway Research Institute, Albany, NY 12208, USA

## Abstract

The polyphenol piceatannol has shown inhibition against tyrosine and serine/threonine kinases. Whether piceatannol also exerts activity on the mammalian target of rapamycin (mTOR), a kinase involved in growth control of eukaryotic cells, is not known. In this study, we tested the effects of piceatannol on proliferation of androgen-dependent (AD) LNCaP and androgen-independent (AI) DU145 and PC-3 prostate cancer (CaP) cells. Suppression of AD and AI CaP cell growth by piceatannol was accompanied by cell cycle blockade in G_1_/S and S phases for LNCaP and PC-3 and induction of apoptosis in DU145 cells. Induction of apoptosis by piceatannol in DU145 cells was evident by reduced expression of poly(ADP-ribose) polymerase (PARP), cleavage of caspase 3 and apoptosis inducing factor AIF, and an increase in cytochrome c. The apoptotic changes occurred in concordance with DNA damage, supported by increased phosphorylated histone H2AX. Immunoblot analyses showed that exposure of different-stage CaP cells to piceatannol also resulted in cell-type-specific downregulation of mTOR and its upstream and downstream effector proteins, AKT and eIF-4E-BP1. We propose that the observed AKT and mTOR changes are new targets of piceatannol possibly contributing to its inhibitory activities on proliferation of CaP cells.

## 1. Introduction

Piceatannol (3,3′,4,5′-tetrahydroxy-trans-stilbene) is a polyphenol found in food sources such as grapes, berries, peanuts, and sugar cane [1–4]. When first isolated from *Euphorbia lagascae*, piceatannol was found to exhibit antileukemic activity [5]. Subsequent experiments showed that piceatannol displays antioxidant [6, 7], anti-inflammatory [8, 9], and anticarcinogenic properties [10–12]. In cell model studies, piceatannol prevented TNF-induced NF*κ*B activation [9, 13, 14], by controlling the oxidoreductive status of cysteine-179 in IKK*β* [4]. Suppression of lung metastasis occurred in Lewis-lung-carcinoma-bearing mice fed piceatannol-fortified diet [15]. 

Mechanistically, piceatannol has been investigated as an inhibitor for tyrosine kinases, including Syk [16–18], FAK [19, 20], and serine/threonine kinases [21]. Furthermore, piceatannol is also used to explore the role of the mitochondrial F_0_F_1_-ATPase [22, 23], in relation to apoptosis. Recent studies show that piceatannol inhibits proliferation and induces cell cycle arrest and apoptosis in DU145 CaP cells [24–26] and that the anticellular effects of piceatannol are mediated by suppression of the cyclin-dependent protein kinase activities (CDKs) [26]. These results suggest that piceatannol might have chemopreventive potential for CaP.

The mTOR is a serine/threonine protein kinase that plays a crucial role in sensing the availability of nutrients for control of cell growth, generally conferring survival benefits [27, 28]. Since mTOR is frequently deregulated in cancer and because, as mentioned, piceatannol acts as a potent kinase inhibitor, it is of interest to determine whether piceatannol might affect mTOR activity/expression and in turn disrupt mTOR-mediated signaling events.

In this study, we tested the hypothesis that piceatannol controls proliferation of both androgen-dependent (AD) and rogen-independent (AI) CaP cells by targeting the expression of mTOR. We also determined whether piceatannol disrupts the mTOR signaling pathway in CaP cells by eliciting changes in mTOR and its upstream and downstream effector proteins: mTOR, protein kinase AKT, initiation factor eIF-4E regulatory binding protein eIF-4E-BP1, and ribosomal protein p70 S6 kinase. We found that piceatannol suppressed AD and AI CaP cell proliferation and that its growth inhibitory activity was accompanied by reduced expression of mTOR and its key effectors AKT and eIF4EBP-1. 

## 2. Materials and Methods

### 2.1. Reagents

Piceatannol was obtained from A.G. Scientific, Inc. (San Diego, CA). The translational control sample kit was from Cell Signaling Technology, Inc. (Beverly, MA). The primary antibodies for cyclins D1 and E, CDKs 2 and 6, AIF, caspase 3, cytochrome c, actin, and secondary antibodies were from Santa Cruz Biotechnology, Inc. (Santa Cruz, CA). The antibody against phosphorylated histone H2AX (Ser139) was from Upstate Biotechnology Inc. (Lake Placid, NY). The antibodies for PARP were from Biomol International, L.P. (Plymouth Meeting, PA). Fetal bovine serum (FBS), RPMI 1640, penicillin, and streptomycin were from Cellgro, Inc (Herndon, VA). All other chemicals and solvents used were of analytical grade.

### 2.2. Cell Culture, Colony Formation, and Proliferation Assays

Human LNCaP, DU145, and PC-3 cells were obtained from the American Tissue Culture Collection (Manassas, VA) and cultured in RPMI 1640 supplemented with penicillin, streptomycin, and 10% heat-inactivated FBS, as described [29]. Piceatannol was dissolved in dimethyl sulfoxide (DMSO) and added to the culture media at the specified dose. Colony formation assay was performed as detailed [30]. Briefly, cells (800–2000 cells/mL, 2 mL/well in 6-well plates) were incubated with increasing doses of piceatannol. Colonies were stained with 1.25% crystal violet, extracted with 10% acetic acid, and quantified by spectrometry at 595 nm. The experiments were performed in triplicate. For cell proliferation assays, cells were seeded in 6-well plates at a density of 1 × 10^5^ cells/mL for LNCaP cells and 5 × 10^4^ cells/mL for DU145 and PC-3 cells. Following treatment, control and treated cells were assayed by trypan blue exclusion using a hemocytometer [29]. Harvested cells were washed with PBS and stored at −80°C for subsequent biochemical analyses.

### 2.3. Cell Cycle Analysis

Cells were treated with 0, 10, and 25 *μ*M piceatannol for 72 h, washed with PBS, and stained with 1.0 *μ*g/mL DAPI (Sigma Chemical Co., St. Louis, MO). Cell cycle phase distribution was assayed by flow cytometry [31, 32]. MultiCycle software program from Phoenix Flow Systems (San Diego, CA) was used to deconvolute the cellular DNA histograms and quantify the percentage of cells in the G_1_, S, and G_2_M phases. Induction of apoptosis was also assayed by flow cytometry, as the sub-G_1 _peak [31, 32].

### 2.4. Preparation of Whole-Cell Extracts and Western Blot Analysis

Cells were collected and lysed in ice-cold RIPA buffer, which contained 50 mM Tris, pH 7.4, 150 mM NaCl, 1 mM EDTA, 1% Triton X-100, 1% deoxycholate, 0.1% SDS, 1 mM dithiothreitol, and 10 *μ*L/mL protease inhibitor cocktail from Sigma-Aldrich Corp. (St. Louis, MO). Protein concentrations of cell lysates were determined by coomassie protein assay kit (Pierce, Rockford, IL) using bovine serum albumin BSA as the standard. For immunoblot analysis, lysates containing 10 *μ*g of protein were separated by 10% SDS-gel electrophoresis, followed by transfer to nitrocellulose membranes and blocked with TBST buffer (10 mM Tris, pH 7.5, 100 mM NaCl, and 0.05% Tween 20) containing 3% nonfat dried milk. The blots were incubated with specific primary antibodies, followed by secondary antibodies. Immunoreactivity was detected by enhanced chemiluminescence (ECL) using the instructions provided by the manufacturer (Kirkegaard & Perry Laboratories, Inc., Gaithersburg, MD). The expression of actin was used as loading control. The intensity of the specific immunoreactive bands was densitometrically quantified and expressed as a ratio relative to the expression of actin.

### 2.5. Data Analysis

The results were expressed as mean ± standard deviation (SD). One-way ANOVA and Student's *t*-test were used to determine the significance of differences in measured variables between control and piceatannol-treated cells. Statistical significance was set at *P* ≤ 0.001.

## 3. Results

### 3.1. Piceatannol-Inhibited Clonogenicity and Proliferation in CaP Cells

**Figure 1:**
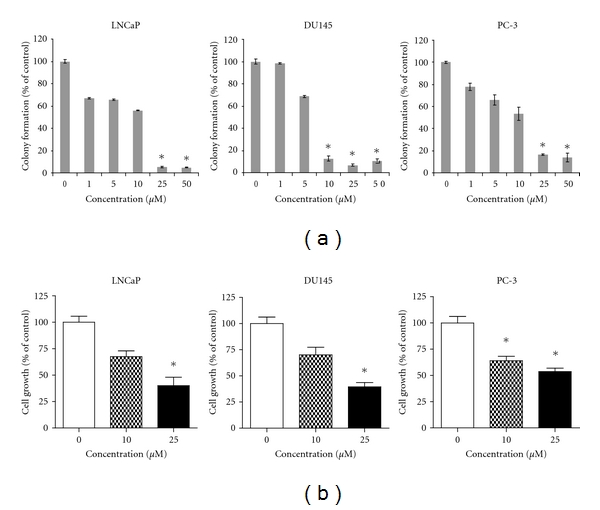
Effects of piceatannol on colony formation and cell proliferation in LNCaP, DU145, and PC-3 CaP cells. (a) Control of clonogenicity by piceatannol. Cells were plated in 6-well tissue culture dishes at 2,000 cells/mL and treated with increasing concentrations of piceatannol (0, 1, 5, 10, 25, and 50 *μ*M). After 1 week, colonies were stained with 1.25% crystal violet and quantified by measuring the absorbance at 595 nm. Dose-dependent suppression of colony formation by piceatannol was expressed as a percentage of control (set as 100%). Values are expressed as mean ± SD for three separate experiments. The symbol represents statistical significance: *P ≤ 0.001. (b) Control of cell growth by piceatannol. Cells were treated with increasing doses of piceatannol (0, 10, and 25 *μ*M). The cell numbers were determined at 72 h using a hemocytometer. The bars show dose-dependent suppression of growth, expressed as a percentage of control (set as 100%). Values are expressed as mean ± SD for three experiments. The symbol represents statistical significance: *P ≤ 0.001.

In previous studies by Kim and coworkers, DU145 cells maintained in DMEM/F12 culture media supplemented with 1% charcoal-treated FBS were followed by 24 h serum deprivation and then treatment by piceatannol; exposure to the polyphenol inhibited cell proliferation and induction of apoptosis [25]. It was of interest to ascertain whether piceatannol exerts a similar antiproliferative activity in CaP cells representing different stages of disease progression without prior serum depletion. Androgen-receptor-(AR)-positive hormone-responsive LNCaP and AR-negative hormone-nonresponsive DU145 and PC-3 CaP cells were exposed to 0, 1, 5, 10, 25, and 50 *μ*M piceatannol, and effects on colony formation were determined. The addition of >10 *μ*M piceatannol caused 50% suppression in foci forming ability in all three cell lines tested (Figure 1(a)). Similarly, as assayed by trypan blue exclusion, a dose-dependent inhibition of proliferation was observed in the three cell lines treated for 72 h with 0, 10, and 25 *μ*M piceatannol (Figure 1(b)).

### 3.2. Piceatannol-Induced Cell Cycle Arrest and Apoptosis in CaP Cells

**Figure 2:**
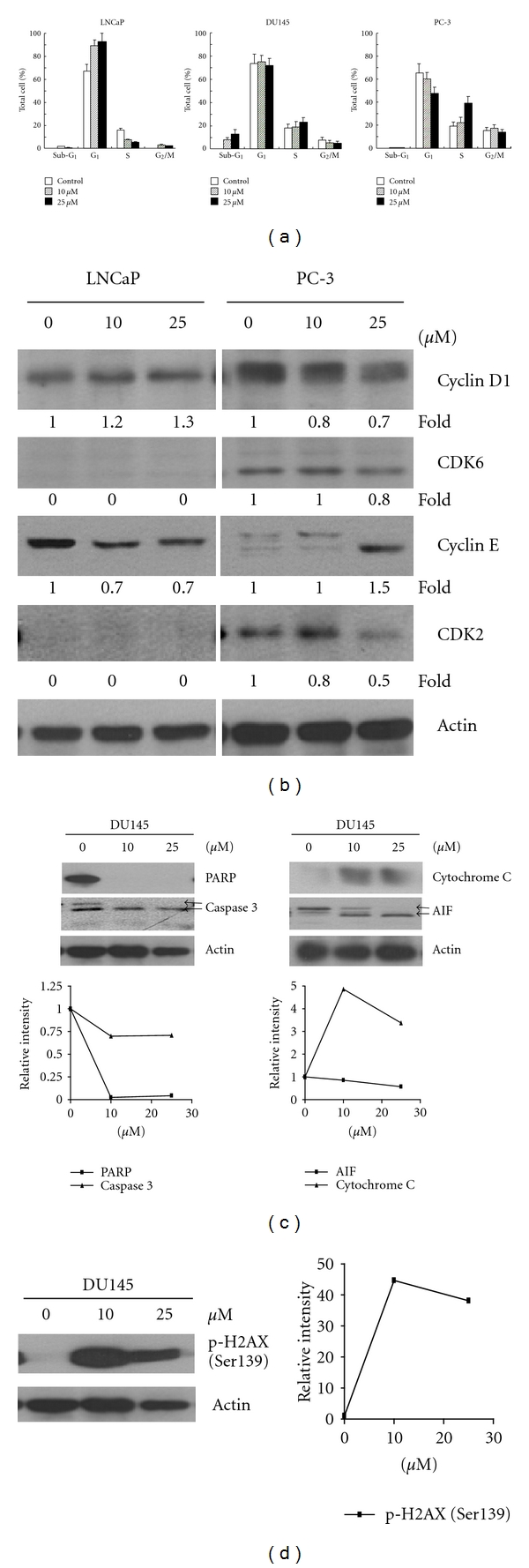
Effects of piceatannol on cell cycle control in LNCaP, DU145, and PC-3 CaP cells. Cells were treated with varying concentrations of piceatannol (0, 10, and 25 *μ*M) for 72 h and changes of cell cycle by piceatannol were further analyzed. (a) The effect on cell cycle distribution was analyzed by flow cytometry. The percentage of cells in G_1_, S, and G_2_M phases were calculated and values are expressed as mean ± SD. Cells with hypodiploid DNA content (sub-G_1_) representing fractions undergoing apoptosis were also calculated. (b) Changes on the expression of various cell cycle regulatory proteins by piceatannol in LNCaP and PC-3 cells. Western blot analysis of cyclins D1 E and CDK6/CDK2 protein expression levels in total cell lysate treated with piceatannol for 72 h. (c) Effects of piceatannol treatment on apoptosis-associated proteins in DU145 cells. Changes in expression of PARP, caspase 3, cytochrome C, and AIF were further analyzed by western blot analysis. (d) Effects of piceatannol on DNA-damage-associated changes in DU145 cells were further analyzed by determining the changes in the expression of phosphorylated H2AX using western blot analysis. Actin expression was used as a loading control. The intensity of the specific immunoreactive bands was quantified by densitometry and expressed as a fold difference against actin.

The nature of growth suppression by piceatannol was next studied by measuring effects on cell cycle distribution by flow cytometry. Exposure to piceatannol resulted in cell-type-dependent changes: (i) LNCaP cells showed G_1_/S arrest, evident by an increase in the G_1_ cell population and a corresponding diminution in S phase cells (Figure 2(a)). (ii) As previously noted [25, 26], a significant induction of apoptosis occurred in DU145 cells (Figure 2(a)), although, without altering cell cycle phase transition. (iii) S phase accumulation, concomitant with reduction in percentage of G_1_ cells, was observed in PC-3 cells (Figure 2(a)).

The differential cell cycle effects elicited by piceatannol in LNCaP and PC-3 cells prompted us to assess the changes on the expression of cell cycle regulatory proteins by western blot analysis. As cyclins D1and E and CDK2/CDK6 play a pivotal role in controlling the cells entry from G_1 _ into the S-phase, the changes on their expression were first measured. In LNCaP cells, increased cyclin D1 but suppressed cyclin E expression with relative low to undetectable expression of CDK2 and CDK6 was found in piceatannol-exposed cells (Figure 2(b)), in partial support of the G_1_ cell arrest (Figure 2(a)). For PC-3 cells, a dose-dependent reduction in cyclin D1/CDK6/CDK2 and induction of cyclin E expression were observed following treatment by piceatannol (Figure 2(b)), consistent with an increase in S paralleled by a decrease in G_1_ phase transition (Figure 2(a)).

To corroborate induction of apoptosis in piceatannol-exposed DU145 cells (Figure 2(a)), the expression of several apoptosis-related genes was assayed. Levels of PARP and pro-caspase 3 were substantially decreased in cells treated for 72 h with 10 and 25 *μ*M piceatannol (Figure 2(c)). Other apoptosis marker changes induced by piceatannol included cleavage of matured 62-kD AIF to its 57-kD product [33] and increase in total cytochrome c (Figure 2(c)). Further analysis implicated DNA damage as a molecular antecedent for piceatannol-induced apoptosis as phosphorylation of Ser139 in histone H2AX, a marker for global DNA damage [34–36], showed a marked elevation in DU145 cells treated with 10 or 25 *μ*M piceatannol (Figure 2(d)).

### 3.3. Piceatannol-Suppressed mTOR Expression, in Concordance with Reduced Levels of eIF4E-BP1 and AKT

**Figure 3:**
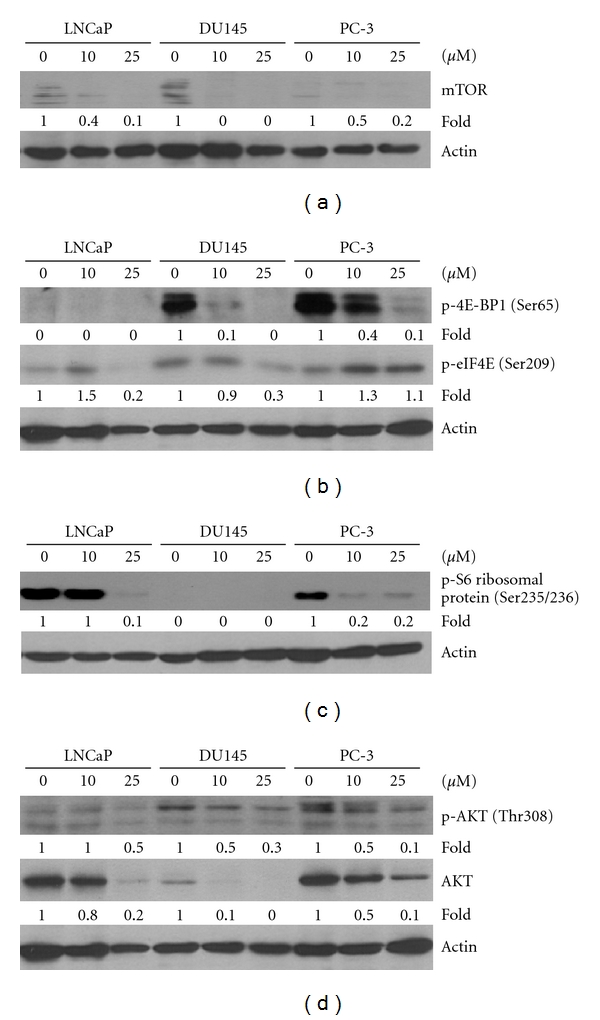
Effects of exposure to piceatannol on mTOR and its downstream p-4E-BP1/p-eIF4E and upstream AKT expression in LNCaP, DU145, and PC-3 cells. Cells were treated with varying concentrations of piceatannol (0, 10, and 25 *μ*M) for 72 h, and immunoblot analysis was used to assess the changes in protein levels of (a) mTOR, (b) phosphorylated p-4E-BP1 (Ser65), and p-eIF4E (Ser209) (c) phosphorylated p-S6 ribosomal protein (Ser235/236), (d) total and phosphorylated AKT (Thr308). In each case, actin was used as a loading control. The intensity of the specific immunoreactive bands was densitometrically quantified and expressed as a fold difference against actin.

To obtain further information on piceatannol-induced growth arrest, we tested the possible involvement of mTOR and mTOR-linked signaling events. A dose-dependent reduction of mTOR expression occurred in all three CaP cells exposed to piceatannol, whereas differential expression of mTOR among three CaP cells was also observed (Figure 3(a)). Piceatannol also affected mTOR downstream effectors eIF-4E and eIF4E-BP1 [37, 38]. For example, a decrease in state of phosphorylation in Ser209 of eIF-4E and in Ser65 of eIF4E-BP1 was observed in DU145 cells (Figure 3(b)), whereas reduced phosphorylated eIF4E (Ser209) was observed in LNCaP with undetectable changes on eIF4E-BP1 (Ser65), following treatment by 25 *μ*M piceatannol (Figure 3(b)). In PC-3 cells, piceatannol caused downregulation of phosphorylated eIF4E-BP1 (Ser65) while slightly increasing phosphorylated eIF4E (Ser209) (Figure 3(b)). We additionally tested if piceatannol affected S6 ribosomal protein levels and phosphorylation of p70-S6 kinase, both involved in control of cell proliferation, functioning as mTOR downstream targets [39, 40]. All three CaP cell lines showed undetectable p70-S6 kinase (Thr389) (data not shown). However, piceatannol inhibited p-S6 (Ser235/236) expression in LNCaP and PC-3 cells (Figure 3(c)). Since AKT is an upstream modulator and activator of mTOR [41, 42], we analyzed changes in AKT expression. A dose-dependent suppression of total and Thr308-phosphorylated AKT was found in all three CaP cells treated by piceatannol (Figure 3(d)).

## 4. Discussion

We have investigated the growth suppressive activities of piceatannol by exposing cultured cells representing different stages of CaP, respectively, LNCaP (AD), DU145 and PC-3 (both AI) to the polyphenol without prior serum depletion. A dose-dependent inhibition of proliferation and clonogenicity was observed in all CaP cells tested; PC-3 cells were more effectively affected using the proliferation assay (Figure 1), while most significant reduction in colony formation was found in DU145 cells (Figure 1). These results confirmed and extended the reported growth control and apoptotic attributes of piceatannol [25, 26] and suggest that this polyphenol inhibits CaP proliferation irrespective of cellular dependency on hormones or culture conditions.

Previously, it has been reported that piceatannol exerts selectivity and potency against DU145 CaP cells [25] compared to normal prostate PWR-1E cells [25, 26] and that it induces cell cycle arrest and apoptosis via the inhibition of CDK activity and activation of the death receptor/mitochondrial-dependent pathways, respectively [25, 26]. To more fully glean its anti-CaP potential, we sought to elucidate further insights into its mechanism of action. In DU145 cell studies we have found that exposure to piceatannol resulted in (i) PARP and caspase 3 changes (Figure 2(c)) implicating the involvement of caspase-dependent mode of cell death, (ii) AIF and cytochrome c changes (Figure 2(c)) implicating the involvement of mitochondria-dependent, caspase-independent mode of cell death, (iii) increased histone H2AX Ser139 phosphorylation (Figure 2(d)) suggesting coupling between induction of apoptosis with piceatannol-elicited DNA damage, and (iv) disruption of mTOR signaling, as evident by downregulation of AKT, mTOR, and eIF-4E-BP1 protein expression. Furthermore, suppression of proliferation and differential downregulation of AKT/mTOR expression were also found in LNCaP and PC-3 cells treated with piceatannol. The demonstration that piceatannol induces marked reduction in AKT and mTOR levels in CaP cells (Figure 3) is significant and suggests a novel mechanism by which this polyphenol acts as a dietary agent for chemoprevention of CaP. Conceivably, piceatannol may affect CaP cell proliferation by targeting protein synthesis, mediated in part through the AKT/mTOR/eIF-4E-BP1 pathway. AKT is robustly activated in various cancers and the AKT signaling cascade is well integrated with growth-factor-mediated pathways in CaP, having implications in CaP survival and development [41, 43]. There is a wealth of evidence in support of the profound role mTOR plays in CaP. For example, inhibitors of the mTOR pathway have been shown to restrict CaP cell proliferation. Furthermore, suppression of mTOR by rapamycin effectively reverses AKT-dependent prostatic intraepithelial neoplasia (PIN) in mouse model studies [43].

**Figure 4:**
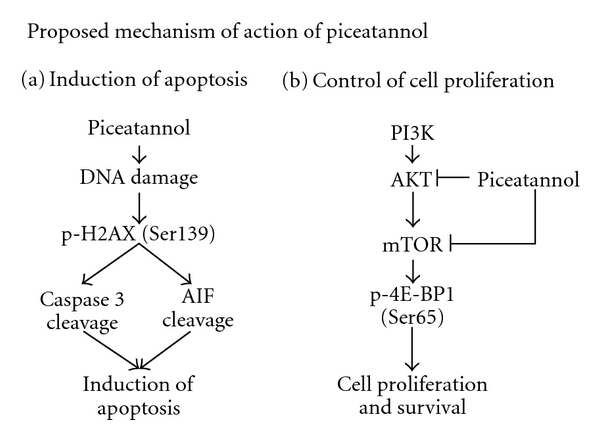
A model mechanism on the ability of piceatannol to induce apoptosis and suppress cell proliferation in CaP cells is proposed. (a) For induction of apoptosis, the effects of piceatannol is postulated to occur secondary to DNA damage accompanied by increased phosphorylation of H2AX at Ser139, which facilitate and culminate in the cleavage of caspase 3 and AIF. (b) for suppression of proliferation, piceatannol is proposed to inhibit the AKT/mTOR signaling pathway resulting in suppression of CaP cell proliferation and survival.

Since mTOR also acts as a molecular sensor and enforcer of cellular responses to changes in growth factors and nutrient status of the cell, by turning on the synthesis of proteins including those critically involved in the control of growth and cell cycle phase transition [44, 45], we assayed changes in one of the downstream translational effectors of mTOR, eIF-4E, and eIF-4E-BP1 known to be involved in mRNA assembly into productive initiation complexes in eukaryotic protein synthesis. Both were found to be modulated by piceatannol, suggesting that this dietary agent acts by regulating the dynamics of interplay between the engagement and sequestration of initiation factors with a dominant role in mRNA binding and translation and in the regulation of specific mRNA recruitment from mRNA-ribonucleoprotein particles for participation in protein synthesis and growth control. One aspect of our current hypothesis is that the mTOR signaling pathway is a potential target of piceatannol and that CaP showing propensity for activation by mTOR might be selectively responsive to treatment by piceatannol.  Figure 4 depicts the proposed anti-CaP mechanism(s) of piceatannol.
